# Society Meetings

**Published:** 1907-06

**Authors:** 


### SOCIETY MEETINGS.

The American Medical Association will hold its sixtieth annual meeting at Atlantic City, June 4-7, 1907. The indications are that this will be more numerously attended than any previous meeting of the association. The president, Dr. William J. Mayo, of Rochester, Minn., will open the meeting at 11 a.m. Tuesday, and among other duties will introduce the president-elect, Dr. Joseph D. Bryant, of New York, and then retire from office.

The Rochester Academy of Medicine held meetings during the month of May as follows:

*Section on Obstetrics, Gynecology, and Pediatrics.*—Friday, May 3, 1907, at 8:15 P.M. Program: Should cholecystitis or cholelithiasis be any longer considered medical affections, and what are the usual consequences of so treating them? Charles B. G. DeNancrede, Professor of Surgery, University of Michigan, Ann Arbor.

The regular quarterly meeting was held Wednesday, May 15, 1907, at 8:15 P.M. Program: Some phases of the cancer problem. William Seaman Bainbridge, New York.

---

THE BUFFALO ACADEMY OF MEDICINE held meetings during the month of May as follows:

*Section on Surgery.*—Tuesday, May 7, 1907, at 8:30 P.M. Program: (a) Urethral stricture, William C. Phelps; (b) How often are vesical calculi overloaded? With a report of ten cases, James A. Gardner. Election of officers of the section.

*Section on Pathology.*—Tuesday, May 21, 1907, at 8:30 P.M. Program: (a) Recent study of neurofibrils, (b) Degeneration of posterior columns in the spinal cord. Illustrated with lantern slides. Charles I. Lambert, of the Pathological Institute for the New York State Hospital, New York city. Election of officers of the section.

*Section on Medicine.*—Tuesday, May 14, 1907, at 8:30 P.M. Program: Symposium on hereditary syphilis: (a) Etiology and general pathology, N. G. Russell; (b) General course and symptoms, Albert T. Lytle; (c) Nose, throat and ear manifestations, George F. Cott; (d) Eye manifestations, Arthur G. Bennett; (e) Skin manifestations, Grover W. Wende. These papers will be illustrated by stereopticon views. Election of officers of the section.

---

THE MEDICAL SOCIETY OF THE COUNTY OF STEUBEN held its regular annual meeting at Bath, May 15, 1907, at which the following named officers were elected for the ensuing year: president, Herbert B. Smith, of Corning; vice-president, F. H. Coyle, of Hornell; secretary, W. W. Smith, of Avoca. Drs. Carpenter of Corning, Wakeman of Hornell, and Ainsworth of Addison were appointed as a committee to confer with the Steuben county board of supervisors relative to the appointment of a county bacteriologist.

---

THE NIAGARA FALLS ACADEMY OF MEDICINE held its annual meeting at the home of Dr. J. A. Lanigan, May 20, 1907. Resolutions upon the death of two members of the academy, Dr. W. A. Campbell and Dr. O. E. McCarty, were read and adopted. The election of officers resulted as follows: President, R. H. Wixon; vice-president, J. A. Lanigan; secretary and treasurer, E. B. Horton; trustee, E. P. H. Griswold. Dr. E. M. Dooley of Buffalo read the paper of the evening.

